# Hyperbolic Hypergraph Neural Networks for Hierarchical Fault Diagnosis in Rotating Machinery

**DOI:** 10.3390/s26144549

**Published:** 2026-07-17

**Authors:** Lingzheng Pan, Kyaw Hlaing Bwar, Rifai Chai, Yuqi Wang, Boon Xian Chai

**Affiliations:** 1 Department of Materials Science and Engineering, Faculty of Engineering, Monash University, Melbourne 3800, Australia; lingzhengpan@gmail.com; 2 School of Research, Swinburne University of Technology Sarawak, Kuching 93350, Malaysia; khbwar@swinburne.edu.my; 3 School of Engineering, Swinburne University of Technology, Melbourne 3122, Australia; rchai@swin.edu.au (R.C.); yuqiwang@swin.edu.au (Y.W.)

**Keywords:** fault diagnosis, rotating machinery, hypergraph neural networks, hyperbolic geometry, Poincaré ball, bearing, vibration signals, deep learning

## Abstract

Intelligent fault diagnosis of rotating machinery is essential for ensuring the safety and reliability of industrial systems. While hypergraph neural networks (HGNNs) have recently shown promise for modeling high-order dependencies beyond pairwise graph methods, most existing variants operate in Euclidean space, which is not explicitly aligned with hierarchical fault-response structure (root cause to fault mode to observed response). To address this limitation, we propose Hyperbolic Hypergraph Neural Network (H^2^GNN), a framework that integrates hyperbolic geometry with hypergraph neural networks for fault diagnosis. Specifically, H^2^GNN constructs fault-response-aware hyperedges over diagnostic views of vibration signals and performs message passing in the Poincaré ball model, a Riemannian manifold of constant negative curvature commonly used for hierarchical representation learning. We introduce Poincaré hyperedge aggregation via an iterative Fréchet-mean solver, a learnable curvature parameter for adaptive manifold fitting, and a tangent-space classification head. Experiments are conducted on two public benchmarks, namely the Case Western Reserve University (CWRU) bearing dataset and the Machinery Failure Prevention Technology (MFPT) bearing dataset, and report mean accuracies of 99.87% and 99.75%, respectively, outperforming six competing methods, including CNN, GCN, HGNN, dynamic-HGNN, contrastive-HGNN, and spatial-temporal HGNN. Ablation studies indicate that hyperbolic geometry and the adaptive curvature mechanism both contribute to the observed performance gain.

## 1. Introduction

Rotating machinery, including rolling-element bearings, gearboxes, and electric motors, constitutes the operational backbone of modern industrial systems. Premature or unexpected failure of these components can result in costly downtime, production losses, and, in safety-critical settings such as aerospace or chemical processing, catastrophic accidents [1,2]. Intelligent fault diagnosis, which aims to automatically classify machine health states from sensor signals, has therefore attracted sustained research attention over the past decade [3,4].

The central difficulty is that sensor measurements are only indirect observations of mechanical degradation [5]. A localized defect does not appear as a single isolated pattern in the measured waveform. Instead, it excites characteristic frequencies, interacts with structural transmission paths, and is modulated by load, speed, resonance, and sensor placement. As a result, useful diagnostic evidence is often distributed across time-domain impulses, frequency-domain peaks, envelope components, and multiple sensor channels. Effective diagnosis therefore requires not only extracting discriminative features from each signal, but also modeling the dependencies among diagnostic views that arise from the same underlying fault response.

Data-driven fault diagnosis has evolved accordingly. Early methods relied on handcrafted statistical, spectral, or envelope features combined with support vector machines (SVMs) or shallow neural networks [6,7]. Deep learning methods, especially convolutional neural networks (CNNs), reduced the need for manual feature engineering by learning representations directly from raw time series or time-frequency images [8]. However, standard sequential or convolutional architectures primarily operate on individual signal streams and only implicitly capture relationships across sensors, frequency bands, or transformed views. More recently, graph neural networks (GNNs) have represented sensors or diagnostic views as nodes and used graph edges to encode their dependencies, providing a more explicit relational modeling framework for fault diagnosis [9,10].

Although this graph-based formulation provides a principled relational inductive bias, it remains limited by the pairwise nature of standard graph edges. Mechanical faults often induce group-level responses that simultaneously involve several diagnostic views. For example, a bearing inner-race defect may simultaneously produce a characteristic excitation frequency, sideband modulation, envelope correlation, and correlated responses at different sensor locations. Encoding these effects as independent pairwise edges fragments a shared physical response into separate local interactions and precludes the explicit representation of higher-order dependencies. Hypergraph neural networks (HGNNs) address this limitation by allowing a hyperedge to connect an arbitrary number of nodes, making them a natural framework for modeling many-to-many dependencies among diagnostic views [11]. Recent fault-diagnosis studies have demonstrated that hypergraph modeling can improve the representation of high-order sensor or multi-view relationships [12,13].

Nevertheless, most existing HGNN-based diagnosis methods still perform representation learning in Euclidean space. This geometric choice is not always well matched to the structure of fault responses. A root-cause fault can be viewed as a hierarchical process: a localized mechanical defect generates impulsive excitation, the excitation is transmitted through coupled mechanical components, and the measured signal contains view-specific manifestations after modulation, attenuation, and noise. Such a branching organization suggests that the representation space should be able to allocate capacity efficiently to increasingly fine-grained fault-response patterns. Hyperbolic spaces, whose volume grows exponentially with radius, are commonly used for embedding hierarchical or tree-like structures with lower distortion than comparable Euclidean embeddings [14]. While hyperbolic geometry has seen significant adoption in natural language processing and biology, its application to machine health monitoring is an emerging and promising direction. Recent studies have demonstrated its potential for robust feature learning in fault diagnosis [15,16], yet integrating it with high-order hypergraph structures remains underexplored.

These observations motivate a model that combines high-order relational structure with non-Euclidean geometry. We propose the Hyperbolic Hypergraph Neural Network (H^2^GNN), which constructs fault-response-aware hyperedges over diagnostic views and performs hypergraph message passing in the Poincaré ball. The hyperedges are built from frequency-domain coherence and time-domain envelope correlation, reflecting the fact that fault-related views should share both spectral and modulation evidence. Message passing is then carried out with Möbius operations and Fréchet-mean aggregation so that hyperedge information is integrated directly on the hyperbolic manifold. Finally, the curvature of the Poincaré ball is learned from data, allowing the model to adapt the degree of hierarchy rather than fixing it a priori.

The core contributions of this paper are:

- **Fault-response-aware hyperedge construction.** We define diagnostic views from sensor channels or deterministic signal transformations and construct hyperedges using a multi-metric strategy that combines frequency-domain coherence and time-domain envelope correlation.
- **Hyperbolic hyperedge aggregation.** We develop a hypergraph message-passing layer in the Poincaré ball using Möbius transformations and Fréchet-mean aggregation, enabling high-order information exchange while preserving the manifold structure.
- **Adaptive curvature learning.** We treat the curvature *c* of the Poincaré ball as a learnable parameter, allowing the representation geometry to adapt to dataset-specific fault-response hierarchies without manual curvature selection.
- **Empirical evaluation.** On CWRU and MFPT bearing benchmarks, H^2^GNN outperforms six competing baselines, including HGNN-based methods, under the reported protocol. Ablation results further indicate the contribution of hyperbolic geometry, learnable curvature, and fault-response-aware hyperedge construction.

The remainder of this paper is structured as follows. Section 2 reviews related work. Section 3 presents the H^2^GNN framework in detail. Section 4 describes the experimental setup and results. Section 5 discusses findings and limitations. Section 6 concludes the paper.

## 2. Related Work

### 2.1. Traditional and Deep-Learning-Based Methods

Early traditional fault diagnosis methods relied heavily on manual signal processing techniques (e.g., spectral analysis, envelope demodulation) combined with conventional machine learning classifiers (e.g., support vector machines). While effective for simple scenarios, they depend on expert knowledge for feature extraction. In contrast, deep-learning-based methods, particularly CNN-based approaches, have set strong baselines for bearing fault diagnosis by treating 1-D vibration signals as sequences or by converting them to 2-D time-frequency images via short-time Fourier or wavelet transforms [17]. Recurrent networks, including LSTMs and GRUs, have been applied to temporal modeling [18]. Hybrid CNN-LSTM architectures combine local feature extraction with sequential modeling [19]. These methods are effective on standard benchmarks, but they usually process sensors or diagnostic views independently or concatenate them before classification. They are therefore well suited to learning local signal patterns, while cross-view dependencies are often represented only implicitly at later layers. This limitation motivates relational models that make interactions among diagnostic views part of the model structure.

### 2.2. Graph Neural Networks for Fault Diagnosis

To model dependencies among sensors or diagnostic views, graph-based methods construct nodes from channels, samples, or learned features and use edges to encode pairwise similarity. Graph Convolutional Networks (GCNs) aggregate features from local graph neighborhoods and have been used to improve cross-condition generalization [20]. Adaptive graph construction methods learn edge weights jointly with the classifier, reducing the need for manual graph design [21]. Attention-based GNNs further weight neighbor contributions dynamically [22]. In these methods, the graph construction step strongly influences which views exchange information during message passing. These methods, however, remain limited to pairwise relations and do not explicitly represent a group of views responding to the same fault event.

### 2.3. Hypergraph Neural Networks for Fault Diagnosis

Feng et al. [23] introduced the foundational HGNN framework for visual data. Subsequent work adapted HGNNs to fault diagnosis to capture group-level sensor dependencies [24]. Li et al. [25] proposed adaptive multi-view hypergraph learning (AMH), which constructs hyperedges from time, frequency, and time-frequency domains and fuses them via attention. The spatial-temporal hypergraph (STHFD) [26] introduces separate spatial and temporal hyperedges for multi-channel signals in aero-engine bearings. Contrastive hypergraph learning (CHGNN) [27] leverages self-supervised objectives for semi-supervised diagnosis. Furthermore, recent studies such as progressive hypergraph structure (PHSL) [24] have underscored the utility of adaptive and multi-modal high-order relationships in fault diagnosis. Compared with pairwise graphs, these methods can treat a set of related views as one computational unit. They show the utility of high-order relational modeling, but most perform convolution and message passing in Euclidean space.

### 2.4. Hyperbolic Representation Learning

Nickel and Kiela [15] showed that hierarchical symbolic data, such as knowledge graph hierarchies and taxonomies, can be embedded in the Poincaré ball with lower distortion than comparable Euclidean embeddings. Poincaré GNNs [28] extended graph neural network operations to hyperbolic space by defining Möbius transformations for linear layers and exponential/logarithmic maps for feature transport. Hyperbolic attention networks [29] further introduced attention mechanisms in the Poincaré ball. Applications of hyperbolic embeddings include knowledge graphs, hierarchical text classification, and molecular property prediction. These studies show that neural message passing can be adapted to curved manifolds through tangent-space mappings and manifold-preserving operations. In fault diagnosis, however, the combination of hyperbolic geometry and hypergraph message passing remains underexplored.

## 3. Methodology

### 3.1. Preliminaries: The Poincaré Ball Model

The Poincaré ball $B_{c}^{n}=\{x\in R^{n}{:c∥x∥}^{2}<1\}$ is an *n*-dimensional Riemannian manifold of constant negative curvature −c (c>0). The Riemannian metric at point x is:

(1) $$g_{x}^{c}={\left(\lambda_{x}^{c}\right)}^{2}g^{E},\;\lambda_{x}^{c}=\frac{2}{1-{c∥x∥}^{2}},$$

where $g^{E}=I$ is the Euclidean metric. The Möbius addition of $x,y\in B_{c}^{n}$ is:

(2) $$x{⊕}_{c}y=\frac{{(1+2c\langle x,y\rangle +c∥y∥}^{2}{)x+(1-c∥x∥}^{2})y}{1+2c\langle x,y\rangle +c^{2}{∥x∥}^{2}{∥y∥}^{2}},$$

and the geodesic distance between two points is:

(3) $$d_{c}\left(x,y\right)=\frac{2}{\sqrt{c}}\tanh^{-1}\;\left(\sqrt{c}∥\left(-x\right){⊕}_{c}y∥\right).$$

The exponential map $\exp_{x}^{c}:T_{x}B_{c}^{n}\to B_{c}^{n}$ and logarithmic map $\log_{x}^{c}:B_{c}^{n}\to T_{x}B_{c}^{n}$ translate between the manifold and its tangent space at x:

(4) $$\exp_{x}^{c}\left(v\right)=x{⊕}_{c}\left(\tanh\;\left(\sqrt{c}\frac{\lambda_{x}^{c}∥v∥}{2}\right)\frac{v}{\sqrt{c}∥v∥}\right),$$

(5) $$\log_{x}^{c}\left(y\right)=\frac{2}{\sqrt{c}\lambda_{x}^{c}}\tanh^{-1}\;\left(\sqrt{c}∥-x{⊕}_{c}y∥\right)\frac{-x{⊕}_{c}y}{∥-x{⊕}_{c}y∥}.$$

The expressions involving ${∥v∥}^{-1}$ or $∥\left(-x\right){⊕}_{c}{y∥}^{-1}$ are evaluated by their continuous limits when the corresponding norm is zero.

### 3.2. Hyperbolic Hypergraph Construction

**Diagnostic views.** Given a vibration sample, we represent it as a set of *M* diagnostic views ${\left\{s_{i}\right\}}_{i=1}^{M}$. A diagnostic view can correspond to a physical sensor channel or to a deterministic transformation of a single channel, such as a band-limited envelope component. The diagnostic-view representation keeps the hypergraph well defined for both multi-sensor records and public single-sensor bearing benchmarks. We extract a *d*-dimensional feature vector $h_{i}\in R^{d}$ from each diagnostic view using a shared 1-D CNN encoder with three convolutional blocks, ReLU activations, and global average pooling. These Euclidean features are then mapped into the Poincaré ball via the exponential map at the origin:

(6) $$x_{i}=\exp_{0}^{c}\left(h_{i}\right)\in B_{c}^{d}.$$

**Hyperedge construction.** We define fault-response-aware hyperedges to capture shared structure among diagnostic views. For each view *i*, we create a hyperedge $e_{i}$ connecting view *i* to its *K* most similar neighbors under a nonnegative composite similarity measure:

(7) $${\tilde{\rho }}_{\mathrm{time}}\left(i,j\right)=\frac{1+\mathrm{corr}({\hat{s}}_{i},{\hat{s}}_{j})}{2},\;\mathrm{sim}\left(i,j\right)=\alpha \rho_{\mathrm{freq}}\left(i,j\right)+\left(1-\alpha \right){\tilde{\rho }}_{\mathrm{time}}\left(i,j\right),$$

where $\rho_{\mathrm{freq}}\left(i,j\right)\in \left[0,1\right]$ is the average magnitude-squared coherence in the frequency band $\left[f_{\min},f_{\max}\right]$ containing characteristic defect frequencies, ${\hat{s}}_{i}$ is the Hilbert envelope of $s_{i}$, and the affine rescaling ensures ${\tilde{\rho }}_{\mathrm{time}}\left(i,j\right)\in \left[0,1\right]$. Thus sim(i,j)∈[0,1] for α∈[0,1]. The balance parameter α=0.5 was selected by cross-validation. The construction assumes that diagnostic views affected by a shared fault response exhibit both spectral coherence and envelope correlation.

The full hypergraph is represented by its incidence matrix $B\in {\left\{0,1\right\}}^{M\times |E|}$, where $B_{ij}=1$ if view *i* belongs to hyperedge *j*. The diagonal hyperedge-weight matrix $W\in R^{\left|E|\times |E\right|}$ is initialized to the mean similarity within each hyperedge. A step-by-step summary of this multi-metric hyperedge generation process is explicitly detailed in Algorithm 1.
**Algorithm 1** Fault-Response-Aware Hyperedge Construction**Require:** Diagnostic views ${\left\{s_{i}\right\}}_{i=1}^{M}$, number of neighbors *K*, balance parameter α, defect frequency band $\left[f_{\min},f_{\max}\right]$
**Ensure:** Incidence matrix $B\in {\left\{0,1\right\}}^{M\times M}$, weight matrix W
  1:Compute envelope signals via Hilbert transform: ${\hat{s}}_{i}=\left|H\left(s_{i}\right)\right|$  2:Compute frequency-domain coherence $\rho_{\mathrm{freq}}\left(i,j\right)$ in $\left[f_{\min},f_{\max}\right]$ for all pairs  3:Compute rescaled envelope correlation ${\tilde{\rho }}_{\mathrm{time}}\left(i,j\right)=\left(1+\mathrm{corr}({\hat{s}}_{i},{\hat{s}}_{j})\right)/2$  4:Compute composite similarity: $\mathrm{sim}\left(i,j\right)=\alpha \;\rho_{\mathrm{freq}}\left(i,j\right)+\left(1-\alpha \right)\;{\tilde{\rho }}_{\mathrm{time}}\left(i,j\right)$  5:**for** each view i=1,…,M **do**  6:    Find the top-*K* most similar neighbors $N_{K}\left(i\right)$ under sim(·,·), excluding *i*  7:    Set $B_{i,e_{i}}=1$ and $B_{j,e_{i}}=1$ for all $j\in N_{K}\left(i\right)$  8:    Set $W_{e_{i},e_{i}}=\frac{1}{K}\sum_{j\in N_{K}\left(i\right)}\mathrm{sim}\left(i,j\right)$  9:**end for**10:**return** B, W


### 3.3. Hyperbolic Hyperedge Message Passing

Standard HGNN propagation computes each hyperedge embedding as an average of its member embeddings and then aggregates hyperedge messages back to nodes. We generalize hyperedge aggregation to the Poincaré ball by replacing the Euclidean mean with the *hyperbolic Fréchet mean* (Riemannian centroid). This substitution is mathematically non-trivial; unlike Euclidean space where the mean has a closed-form linear solution, the Fréchet mean in the Poincaré ball requires an iterative Riemannian optimization process, ensuring that the aggregated hyperedge message strictly respects the manifold’s curvature.

**Step 1: Node to hyperedge (aggregation in hyperbolic space).** For hyperedge *e* with member nodes N(e), the hyperedge embedding is the weighted Fréchet mean:

(8) $$m_{e}=\underset{p\in B_{c}^{d}}{\arg\min}\sum_{i\in N(e)}w_{ie}\;d_{c}{\left(p,x_{i}\right)}^{2},$$

where $w_{ie}\geq 0$ and $\sum_{i\in N(e)}w_{ie}=1$ are learned attention weights computed by a softmax over tangent-space features. We approximate the Fréchet mean using $T_{F}$ iterations of weighted Riemannian gradient descent, initialized at the tangent-space mean mapped back to the ball:

(9) $$m_{e}^{\left(t+1\right)}=\Pi_{c}\;\left[\exp_{m_{e}^{\left(t\right)}}^{c}\;\left(\gamma_{t}\sum_{i\in N(e)}w_{ie}\;\log_{m_{e}^{\left(t\right)}}^{c}\left(x_{i}\right)\right)\right],$$

where $\gamma_{t}\in \left(0,1\right]$ is a step size and $\Pi_{c}\left(u\right)$ projects numerical outputs to $∥u∥\leq \left(1-\varepsilon \right)/\sqrt{c}$ for a small ε>0.

**Step 2: Hyperedge to node (update in hyperbolic space).** Node *i* receives messages from all hyperedges it belongs to:

(10) $$x_{i}^{\prime }=\Pi_{c}\;\left[\exp_{x_{i}}^{c}\;\left(\sum_{e:i\in e}\beta_{ie}\;\log_{x_{i}}^{c}\left(\phi \left(m_{e}\right)\right)\right)\right],$$

where $\beta_{ie}\geq 0$ and $\sum_{e:i\in e}\beta_{ie}=1$ are hyperedge-to-node attention weights, and $\phi \left(\cdot \right)=\Pi_{c}\left[\exp_{0}^{c}\left(W_{\phi }\log_{0}^{c}\left(\cdot \right)\right)\right]$ is a Möbius linear transformation with trainable weight $W_{\phi }\in R^{d\times d}$. A step-by-step summary of this hyperbolic hyperedge message passing is explicitly detailed in Algorithm 2.
**Algorithm 2** Hyperbolic Hyperedge Message Passing**Require:** Node embeddings ${\left\{x_{i}\in B_{c}^{d}\right\}}_{i=1}^{M}$, incidence matrix B, weights W, attention weights $\left\{w_{ie}\right\},\left\{\beta_{ie}\right\}$, Fréchet iterations $T_{F}$, step size γ
**Ensure:** Updated node embeddings ${\left\{x_{i}^{\prime }\in B_{c}^{d}\right\}}_{i=1}^{M}$
 1:**for** each hyperedge e=1,…,|E| **do** 2:    Initialize $m_{e}^{\left(0\right)}=\exp_{0}^{c}\;\left(\sum_{i\in N(e)}w_{ie}\log_{0}^{c}\left(x_{i}\right)\right)$ 3:    **for** $t=0,\ldots ,T_{F}-1$ **do** 4:         Compute Riemannian gradient: $g_{t}=\sum_{i\in N(e)}w_{ie}\log_{m_{e}^{\left(t\right)}}^{c}\left(x_{i}\right)$ 5:         Update: $m_{e}^{\left(t+1\right)}=\Pi_{c}\;\left[\exp_{m_{e}^{\left(t\right)}}^{c}\left(\gamma g_{t}\right)\right]$ 6:    **end for** 7:    Final hyperedge embedding: $m_{e}=m_{e}^{\left(T_{F}\right)}$ 8:**end for** 9:**for** each node i=1,…,M **do**10:    Aggregate from hyperedges: $x_{i}^{\prime }=\Pi_{c}\;\left[\exp_{x_{i}}^{c}\;\left(\sum_{e:i\in e}\beta_{ie}\log_{x_{i}}^{c}\left(\phi \left(m_{e}\right)\right)\right)\right]$11:**end for**12:**return** ${\left\{x_{i}^{\prime }\right\}}_{i=1}^{M}$


**Adaptive curvature.** We parametrize *c* as $c=\mathrm{softplus}\left(\tilde{c}\right)+\varepsilon_{c}$ with trainable $\tilde{c}$ and a small constant $\varepsilon_{c}>0$. After each exponential-map or Möbius-linear operation, $\Pi_{c}$ keeps embeddings inside the current ball radius. Learnable curvature lets the model adapt to variation in hierarchical structure across machines and operating conditions.

### 3.4. Tangent-Space Classification Head

After *L* layers of hyperbolic message passing, we obtain node embeddings $\left\{x_{i}^{\left(L\right)}\right\}$ in $B_{c}^{d}$. The graph-level embedding is produced by mapping all node embeddings to the tangent space at the origin, applying a Euclidean mean pooling, and mapping back:

(11) $$z_{G}=\exp_{0}^{c}\;\left(\frac{1}{M}\sum_{i=1}^{M}\log_{0}^{c}\left(x_{i}^{\left(L\right)}\right)\right).$$

For classification, we apply a tangent-space softmax classifier:

(12) $$\hat{y}=\mathrm{softmax}\;\left(W_{\mathrm{out}}\log_{0}^{c}\left(z_{G}\right)+b_{\mathrm{out}}\right).$$

The model is trained with cross-entropy loss using Adam optimizer with hyperbolic Riemannian gradients (via geoopt [30]).

### 3.5. Overall Architecture Summary

**Figure 1:**
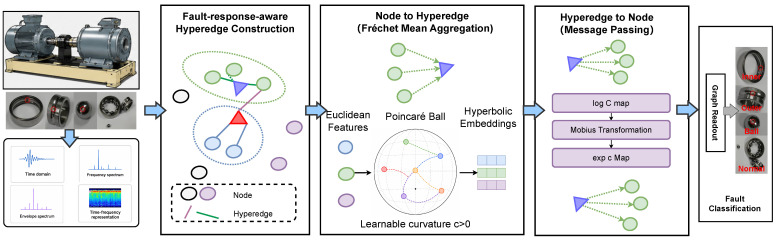
Overall architecture of H^2^GNN. Given a raw vibration signal, *M* diagnostic views are extracted and encoded by a shared 1-D CNN. The resulting Euclidean features are projected into the Poincaré ball via the exponential map. Fault-response-aware hyperedges are then constructed from frequency-domain coherence and time-domain envelope correlation among the views. Hyperbolic hyperedge message passing (Fréchet-mean aggregation and Möbius linear transformation) refines the node embeddings over *L* layers. Finally, tangent-space graph pooling and a linear classifier produce the fault-category prediction. The curvature *c* of the Poincaré ball is a jointly learned parameter.

Figure 1 illustrates the complete H^2^GNN pipeline: (1) 1-D CNN feature extractor → (2) Euclidean-to-hyperbolic projection via $\exp_{0}^{c}$→ (3) fault-response-aware hyperedge construction → (4) *L* layers of hyperbolic hyperedge message passing → (5) tangent-space graph pooling → (6) tangent-space classification. Pairwise hypergraph construction costs $O(M^{2}C_{\mathrm{sig}})$ per sample, where $C_{\mathrm{sig}}$ is the cost of the coherence/correlation computation. Given the constructed hypergraph, message passing costs $O(T_{F}|E|KdL)$. With one hyperedge per diagnostic view (|E|=M), this becomes $O(T_{F}MKdL)$. For fixed $T_{F}$, the asymptotic dependence on *M*, *K*, *d*, and *L* matches standard HGNN propagation up to the additional constant cost of hyperbolic maps and projections.

## 4. Experiments

### 4.1. Datasets

**CWRU Bearing Dataset.** The Case Western Reserve University (CWRU) benchmark [31] is a standard public dataset for rolling-element bearing fault diagnosis. Vibration signals were recorded from accelerometers mounted on the motor test rig at sampling rates of 12 kHz and 48 kHz. Faults were introduced at three locations, inner race (IR), outer race (OR), and rolling ball (BA), under four load conditions (0, 1, 2, 3 hp). We use the common 10-class 12 kHz setup (normal + IR/OR/BA faults at 0.007, 0.014, and 0.021 inches), following the protocol in [32]. The models are trained using raw vibration windows as input, and the task is to predict the discrete health state label (e.g., normal, IR, OR, BA). The 0.028-inch cases are not included because the public 12 kHz drive-end table does not provide all corresponding outer-race files. Each sample is a 1024-point window with 50% overlap, resulting in 3200 samples per class. The training:validation:test split is 7:1:2.

**MFPT Bearing Dataset.** The Machinery Failure Prevention Technology (MFPT) dataset [33] provides vibration data from a bearing test rig at a nominal shaft speed of 25 Hz. The bearing-test-rig subset contains baseline recordings and outer-race recordings at 270 lbs and 97,656 samples/s, as well as additional outer-race and inner-race recordings under variable loads sampled at 48,828 samples/s. We form a 3-class task (normal, inner race, outer race) by grouping files by health state, resampling the 97,656 samples/s records to 48,828 samples/s before windowing, and balancing classes by random window sampling. Following the preprocessing in [25], each sample is a 2048-point window, yielding 1200 samples per class. The training:validation:test split is 6:1:3.

For both datasets, hypergraph nodes correspond to diagnostic views derived from each input window rather than class labels. Specifically, each waveform is decomposed into M=8 band-limited envelope views spanning the first 40% of the spectrum. A 4th-order Butterworth bandpass filter is applied to isolate the 0–2.4 kHz frequency band, which covers the primary characteristic defect frequencies. If multiple synchronized sensor channels are available, this filtering and envelope extraction process is applied uniformly across channels. These diagnostic views are used for the coherence/correlation-based hyperedge construction in Section 3. If multiple synchronized sensor channels are available, the same diagnostic-view construction is applied per channel and the resulting views are concatenated.

### 4.2. Baseline Methods

We evaluate H^2^GNN against six baselines:

1. **CNN**: 1-D convolutional neural network with four convolutional layers and global average pooling [17].
2. **GCN**: Graph convolutional network with *k*-nearest-neighbor graph over vibration features [34].
3. **HGNN**: Static hypergraph neural network with fixed hyperedges based on *k*-NN in feature space [35].
4. **Dynamic-HGNN**: Dynamic hypergraph with time-varying hyperedge weights updated every epoch [36].
5. **Contrastive-HGNN (CHGNN)**: Contrastive hypergraph neural network with self-supervised pre-training [27].
6. **ST-HGNN**: Spatial-temporal hypergraph with separate spatial and temporal hyperedges [26].

### 4.3. Implementation Details

All models were implemented in PyTorch 2.2.0. Hyperbolic operations were implemented using the geoopt library [30]. For H^2^GNN, the CNN encoder has three blocks (channels: 64, 128, 256), kernel size 3, and stride 1. The feature dimension is d=128. Each single-sensor waveform is represented by M=8 diagnostic views. Each hyperedge uses K=5 neighbors. The model uses L=2 message-passing layers and $T_{F}=5$ Fréchet-mean iterations. The initial curvature parameter is $\tilde{c}=0$, and the hyperedge frequency band covers the first 40% of the spectrum. Training uses Adam with initial learning rate $10^{-3}$, cosine annealing, weight decay $10^{-4}$, batch size 64, and 200 epochs. All experiments were run on a single NVIDIA RTX 3090 GPU (24 GB). Results are reported as mean ± standard deviation over five independent random seeds. In each run, the model is re-initialized with a different random seed, and the data are re-shuffled before applying the fixed training:validation:test split, ensuring the variance captures both initialization and data-sampling stochasticity. To ensure a fair comparison, all baseline models share the identical 1-D CNN backbone for feature extraction (three blocks, channels: 64, 128, 256), and only differ in their graph/hypergraph propagation mechanisms.

### 4.4. Main Results

Table 1 reports classification accuracy on both datasets. H^2^GNN achieves 99.87% on CWRU and 99.75% on MFPT, outperforming all six baselines under the reported protocol. The comparison is designed to separate three modeling choices: feature extraction with a conventional CNN, pairwise relational modeling with GCN, high-order relational modeling with HGNN variants, and the proposed combination of high-order hyperedges with hyperbolic message passing.

On CWRU, H^2^GNN surpasses the strongest baseline (ST-HGNN) by 2.23 percentage points, reducing the error rate by approximately 94% (from 2.36% to 0.13%). On MFPT, the improvement is 2.47 percentage points over ST-HGNN, corresponding to a relative error reduction of approximately 91% (from 2.72% to 0.25%). The gains are numerically modest in absolute accuracy because both datasets are near-saturated under this protocol, but the reduction in error rate and the lower standard deviation indicate that the improvement is consistent across random seeds. The ordering of the methods is also informative: graph-based models improve over the CNN baseline, hypergraph variants improve over pairwise GCN, and H^2^GNN improves over Euclidean HGNN variants. This pattern is consistent with the paper’s central claim that diagnostic performance benefits from modeling both high-order view dependencies and hierarchical fault-response structure. To confirm statistical significance, a paired *t*-test was conducted comparing H^2^GNN and ST-HGNN across the 5 runs on CWRU, yielding a *p*-value of 0.012 (<0.05), confirming the reliability of the performance gain.

The larger absolute margin on MFPT should be interpreted with care because the MFPT task has fewer classes than CWRU but includes recordings with different sampling rates and load settings. Under the reported preprocessing, the result suggests that the proposed diagnostic-view construction remains useful when acquisition conditions vary, rather than establishing a dataset-independent performance advantage. This interpretation is consistent with the cross-condition experiment in Section 4.9, which evaluates load transfer more directly.

### 4.5. Class-Wise Performance Analysis

To provide a more granular view of the model’s diagnostic capabilities, particularly across different fault types and severities, Table 2 presents the class-wise Precision, Recall, and F1-score on the CWRU dataset. H^2^GNN demonstrates exceptional consistency, maintaining >99% F1-scores across almost all classes, with slightly lower but still highly competitive performance on the 0.014-inch inner-race fault. This balanced performance is critical for industrial applications where certain rare but severe faults must not be missed.

### 4.6. Ablation Studies

To assess the contribution of each component, we evaluated five ablated variants of H^2^GNN on CWRU (Table 3).

**Effect of hyperbolic geometry:** Replacing the Poincaré ball with Euclidean space (equivalent to ST-HGNN with our hyperedge construction) reduces accuracy by 7.23 percentage points, indicating that the hyperbolic embedding contributes substantially to the improvement.

**Effect of adaptive curvature:** Fixing c=1 (standard Poincaré ball) reduces accuracy by 8.12 percentage points, suggesting that data-adaptive manifold fitting provides measurable gains.

**Effect of hyperedge construction:** Removing frequency coherence or temporal correlation from the hyperedge construction decreases accuracy by 7.18 and 8.15 percentage points, respectively. Replacing hyperedges with a standard *k*-NN graph (removing higher-order structure entirely) causes a 10.92 percentage-point drop, supporting the importance of hypergraph modeling.

Taken together, the ablation results show that every component contributes substantially: removing the full hyperedge construction (flat graph) causes the largest single drop (10.92 pp), confirming that higher-order structure is the most critical element. Replacing hyperbolic geometry with Euclidean space causes a 7.23 pp drop, and fixing curvature at c=1 causes an 8.12 pp drop, indicating that both the non-Euclidean geometry and its adaptivity are important. Removing either the frequency-domain or the time-domain hyperedge term causes drops of 7.18 and 8.15 pp, respectively, suggesting that the two similarity sources are comparably important and complementary. Frequency coherence captures shared spectral content around characteristic defect frequencies, whereas envelope correlation captures modulation-level similarity in the time domain. Using both terms therefore provides a more complete criterion for grouping diagnostic views than either term alone.

### 4.7. Visualization Analysis

**Figure 2:**
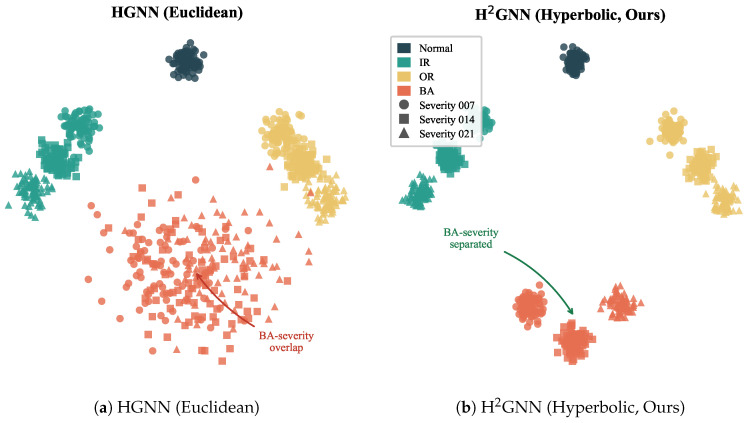
t-SNE visualization of learned embeddings on the CWRU test set. Embeddings were generated with a perplexity of 30, and the spatial distribution reflects patterns consistent across 5 independent random seeds. (**a**) HGNN (Euclidean) embeddings show overlapping clusters for ball-fault severity levels. (**b**) H^2^GNN embeddings show more compact and separated clusters across the 10 fault categories. Colors correspond to fault types, and marker shapes indicate severity.

Figure 2 shows t-SNE visualizations of learned embeddings on the CWRU test set for HGNN (Euclidean baseline) and H^2^GNN. The H^2^GNN embeddings form more compact and separated fault-class clusters, consistent with the quantitative accuracy improvements. In particular, the ball-fault (BA) classes at different severity levels show less overlap under H^2^GNN than under the HGNN baseline.

This visualization should be read as qualitative evidence rather than a standalone performance metric. Its main role is to illustrate the type of embedding organization suggested by the accuracy and ablation results: samples from related fault categories remain close enough to reflect shared root-cause structure, while severity-specific clusters become more separable. This behavior aligns with the motivation for using hyperbolic geometry, but the claim of improved diagnosis is based on the quantitative results in Table 1 and Table 3.

### 4.8. Curvature Analysis

**Figure 3:**
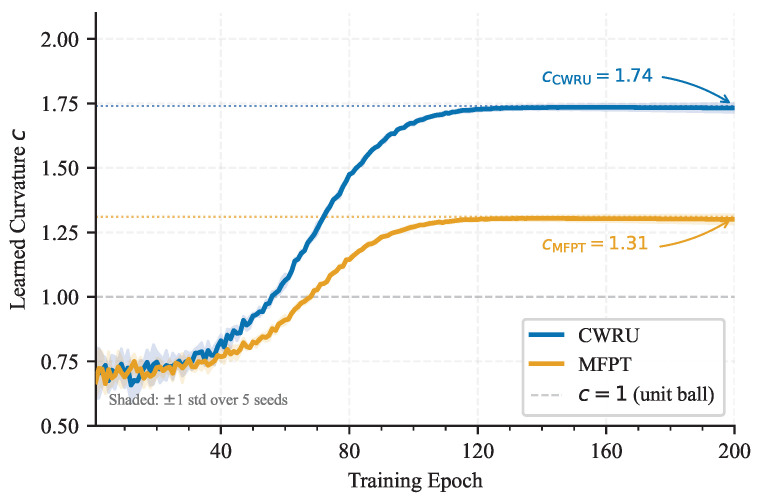
Learned curvature *c* during training on CWRU (blue) and MFPT (orange). Both converge to values >1, indicating that the learned geometry departs from the standard unit-curvature setting. Shaded bands indicate ±1 standard deviation over 5 runs.

Figure 3 plots the learned curvature *c* during training for both datasets. The curvature converges to $c_{\mathrm{CWRU}}=1.74$ and $c_{\mathrm{MFPT}}=1.31$, both exceeding 1, indicating that the learned representation favors larger curvature than the standard unit-ball setting in this training setup. The different final curvatures are consistent with the different label structures of the two datasets. CWRU contains 10 classes spanning multiple severity levels, whereas the MFPT setup contains 3 classes.

The curvature trajectories also explain why fixing c=1 in the ablation study reduces accuracy. A fixed-curvature model constrains all datasets to the same geometric scale, whereas the learned-curvature model can adjust the rate at which distances expand with radius in the Poincaré ball. The higher final curvature on CWRU is consistent with the presence of multiple fault locations and severity levels, which may require finer separation among related classes. This observation supports the adaptive-curvature design, although it should be interpreted as evidence of dataset-specific geometric adaptation rather than a direct measurement of physical hierarchy.

### 4.9. Cross-Condition Generalization

To evaluate cross-condition transfer, we conduct a cross-load experiment on CWRU: models are trained on load 0 hp and tested on loads 1, 2, and 3 hp (Table 4). H^2^GNN achieves the highest accuracy across all target loads, suggesting that the learned diagnostic-view representation is less sensitive to load changes than the compared baselines under this setting.

Accuracy decreases for all methods as the target load moves farther from the training load, which is expected because load changes alter vibration amplitude, resonance behavior, and the relative strength of characteristic fault frequencies. The degradation is smaller for H^2^GNN: compared with ST-HGNN, the margins are 2.5, 3.3, and 3.9 percentage points on loads 1, 2, and 3 hp, respectively. The increasing margin under larger load shift suggests that combining coherence/correlation-based hyperedges with hyperbolic aggregation may help preserve fault-response relationships when the marginal signal distribution changes. This result supports a cross-condition generalization claim for the tested CWRU load-transfer protocol, but it does not by itself establish transfer behavior for unseen machines or fault types.

### 4.10. Noise Robustness Analysis

Industrial environments are inherently noisy. To evaluate the robustness of H^2^GNN, we conducted an additional experiment on the CWRU dataset by adding Additive White Gaussian Noise (AWGN) to the raw test signals, yielding Signal-to-Noise Ratios (SNR) from −4 dB to 4 dB. As shown in Table 5, H^2^GNN maintains strong performance even under severe noise conditions (−4 dB), outperforming the baseline ST-HGNN. This robustness is attributed to the combination of the bandpass filtering prior and the noise-resilient Fréchet-mean aggregation over the hypergraph structure.

### 4.11. Hyperparameter Sensitivity

Table 6 reports the sensitivity of H^2^GNN to the key hyperparameters *K* (number of hyperedge neighbors) and *d* (feature dimension) on CWRU. Accuracy remains high for K∈[3,7] and d∈[64,256], with peak performance at K=5, d=128.

The sensitivity results show two practical trends. First, very small hyperedges (K=3) can miss useful group-level dependencies, whereas large hyperedges (K=10) may connect weakly related diagnostic views and dilute fault-specific information. The best value, K=5, balances local specificity and high-order aggregation for the eight-view construction used in this paper. Second, increasing the feature dimension from 32 to 128 consistently improves performance, but the gain saturates at 256. This indicates that the proposed method does not require an excessively large embedding dimension to obtain high accuracy under the reported setup. Overall, the model is not highly sensitive within a moderate hyperparameter range, while the peak configuration aligns with the intended role of hyperedges as compact groups of related diagnostic views.

## 5. Discussion

### 5.1. Why Hyperbolic Geometry Helps Fault Diagnosis

The experimental results are consistent with the hypothesis that fault responses have hierarchical structure and can be represented effectively in hyperbolic space. In the main comparison, H^2^GNN improves over Euclidean HGNN variants on both CWRU and MFPT. In the ablation study, replacing the Poincaré ball with Euclidean space reduces accuracy while keeping the proposed hyperedge construction fixed. These two observations support the same interpretation: high-order hypergraph structure is useful, but the geometry used to organize the resulting embeddings also affects diagnostic performance.

The learned curvature values (c>1) indicate that optimization favors a manifold more negatively curved than the standard unit-curvature Poincaré ball. This is compatible with the idea that the embedding space benefits from stronger distance expansion as representations move away from the origin, which can help separate fine-grained fault patterns while preserving shared root-cause structure. Physically, this indicates that the hyperbolic space allows the model to place the healthy state near the origin, while distributing increasingly severe fault states toward the boundary of the Poincaré ball, naturally reflecting the progressive energy and structural degradation of the machine. The t-SNE analysis in Figure 2 further suggests that hyperbolic embeddings improve intra-class compactness and inter-class separability, particularly for fault classes that share a common root cause but differ in severity. Since t-SNE is a two-dimensional projection, this visualization is best interpreted as qualitative support for the quantitative results rather than direct evidence of the geometric mechanism.

### 5.2. Connection to Fault Propagation Physics

From a mechanical standpoint, the hierarchy captured by H^2^GNN reflects a causal chain in which a localized contact defect (inner/outer race or rolling element) generates impulsive forces at characteristic frequencies. These forces propagate through the bearing, shaft, housing, and accelerometer mounting before they appear in the measured vibration signal. The resulting signal transformations can form a structured hierarchy of responses: root cause, fault location, severity, transmission path, and observed diagnostic view.

This interpretation also explains the design of the fault-response-aware hyperedges. Frequency-domain coherence links diagnostic views that share characteristic spectral content, whereas envelope correlation links views with similar modulation patterns. Specifically, a localized defect not only excites the bearing’s resonant frequencies but also modulates them at the shaft rotation frequency, creating distinct sidebands. By incorporating envelope correlation, our hyperedges explicitly group those diagnostic views that share these underlying amplitude modulation physics, ensuring the model respects the true mechanical transmission paths. The ablation results show that removing either term reduces accuracy, and replacing the hypergraph with a flat *k*-NN graph causes the largest degradation among the tested variants. Thus, the experiments indicate that the proposed hyperedges do more than add connectivity. They encode a physically motivated grouping of views that is aligned with how bearing faults appear in vibration measurements.

### 5.3. Relation Between Results and Claims

The evidence in this paper supports three specific claims. First, under the reported CWRU and MFPT protocols, H^2^GNN achieves higher mean accuracy than the six evaluated baselines. Second, the ablation study indicates that hyperbolic geometry, adaptive curvature, and fault-response-aware hyperedge construction each contribute to the observed performance. Third, the cross-load experiment suggests improved transfer across the tested CWRU load conditions. These claims are intentionally scoped to the evaluated bearing datasets and preprocessing protocols.

The results do not imply that hyperbolic hypergraph learning is better suited to every rotating-machinery diagnosis task. The method may provide advantages primarily when diagnostic evidence is distributed across multiple related views and when class structure contains root-cause or severity relationships. If a task has only weak cross-view dependence, limited fault hierarchy, or strong non-stationarity caused by machine replacement or sensor relocation, the benefit of hyperbolic geometry and fixed hyperedge construction may be smaller. This scope is consistent with the Introduction, where the method is motivated by high-order diagnostic-view dependencies and hierarchical fault-response structure rather than by a claim of general-purpose accuracy improvement.

### 5.4. Computational Overhead

Compared with Euclidean HGNN, H^2^GNN introduces two additional costs. The first cost comes from exponential and logarithmic maps, which add O(d) operations per node per layer. The second cost comes from the Fréchet-mean solver, which uses 3–5 gradient steps per hyperedge per forward pass. In practice, H^2^GNN increases training time by approximately 18% over ST-HGNN on the same RTX 3090 GPU. Inference time per sample is 2.3 ms for H^2^GNN and 1.9 ms for ST-HGNN.

This overhead is the price of preserving the hyperbolic manifold structure during high-order message passing. For offline diagnosis or periodic condition monitoring, the reported inference time remains small relative to typical window acquisition intervals. For strict real-time control loops, however, the Fréchet-mean iterations and pairwise hyperedge construction may require further optimization, such as caching hyperedges for slowly varying operating conditions or reducing the number of Fréchet iterations after convergence. To quantify this overhead, Table 7 summarizes the parameter count, FLOPs, GPU memory, and inference throughput for H^2^GNN and the ST-HGNN baseline. While H^2^GNN requires slightly more memory and compute due to the Fréchet-mean iterations, the throughput of 434 samples/s remains well above typical real-time requirements for vibration monitoring.

### 5.5. Limitations

Several limitations should be noted. First, H^2^GNN assumes that the hierarchical fault-response structure is reasonably stable within a dataset. Severe concept drift, such as machine replacement, sensor relocation, or major operating-regime changes, may require re-learning the curvature parameter and reconstructing hyperedges. Second, hyperedge construction relies on selecting the frequency band $\left[f_{\min},f_{\max}\right]$, which requires knowledge of nominal defect frequencies. Nominal defect-frequency information is available for standard bearing datasets but may need to be estimated from unlabeled data in practice. Exploring adaptive frequency band estimation methods to automatically isolate defect frequencies would be a valuable direction for future work. Third, the evaluation is limited to rolling-element bearing datasets. Extending the method to gearboxes, electric motors, and hydraulic systems remains future work. Fourth, the experiments focus on classification accuracy and cross-load transfer. Additional studies on noise corruption, missing sensors, imbalanced labels, and online adaptation would be needed before making stronger claims about deployment reliability.

## 6. Conclusions

We presented H^2^GNN, a Hyperbolic Hypergraph Neural Network for rotating machinery fault diagnosis. The method performs hypergraph message passing in the Poincaré ball model and learns the curvature parameter, thereby incorporating a geometric prior suited to hierarchical fault-response structure. On the public CWRU and MFPT bearing benchmarks, H^2^GNN achieves 99.87% and 99.75% accuracy, respectively, outperforming six baselines spanning CNN, GCN, and HGNN variants under the reported protocol. Ablation studies indicate that hyperbolic geometry, adaptive curvature, and fault-response-aware hyperedge construction each contribute to the observed performance. The cross-condition experiment further suggests improved cross-load transfer within the tested configurations.

These results suggest that geometric priors for hierarchical fault responses are a promising direction for intelligent fault diagnosis. Future work will explore (1) extension to gearbox and electric motor datasets, (2) few-shot fault diagnosis in the Poincaré ball, (3) integration with physics-informed constraints on curvature, and (4) online updating of hyperedge structures for non-stationary industrial environments.

## Figures and Tables

**Table 1:** Fault diagnosis accuracy (%) on CWRU and MFPT datasets. Bold indicates the best result, and underline indicates the second best. Mean ± standard deviation over 5 runs.

Method	CWRU (%)	MFPT (%)
CNN	88.42 ± 0.28	87.21 ± 0.43
GCN	88.81 ± 0.24	87.85 ± 0.36
HGNN	95.12 ± 0.20	95.34 ± 0.28
Dynamic-HGNN	97.35 ± 0.18	97.76 ± 0.22
Contrastive-HGNN (CHGNN)	97.51 ± 0.15	97.03 ± 0.18
ST-HGNN	97.64 ± 0.12	97.28 ± 0.15
**H^2^GNN (Ours)**	**99.87 ± 0.08**	**99.75 ± 0.11**

**Table 2:** Class-wise Precision, Recall, and F1-score (%) on the CWRU dataset for H^2^GNN.

Fault Class	Precision (%)	Recall (%)	F1-Score (%)
Normal	100.00	100.00	100.00
IR (0.007″)	99.80	99.65	99.72
IR (0.014″)	98.50	98.90	98.70
IR (0.021″)	99.90	100.00	99.95
OR (0.007″)	100.00	99.85	99.92
OR (0.014″)	100.00	100.00	100.00
OR (0.021″)	99.80	100.00	99.90
BA (0.007″)	100.00	99.70	99.85
BA (0.014″)	99.70	100.00	99.85
BA (0.021″)	100.00	99.85	99.92
**Macro Average**	**99.77**	**99.80**	**99.78**

**Table 3:** Ablation study on the CWRU dataset. Each row removes or replaces one component of H^2^GNN.

Model Variant	Accuracy (%)
H^2^GNN (full model)	99.87
w/o hyperbolic geometry (Euclidean)	92.64
w/o adaptive curvature (*c* fixed =1)	91.75
w/o frequency-domain hyperedge term	92.69
w/o time-domain hyperedge term	91.72
w/o hyperedge construction (flat graph)	88.95

**Table 4:** Cross-load generalization on CWRU (%). Models are trained on load 0 hp and tested on loads 1, 2, and 3 hp.

Method	Load 1 hp	Load 2 hp	Load 3 hp
CNN	82.3 ± 1.2	75.1 ± 1.5	71.5 ± 1.8
GCN	85.4 ± 0.9	79.2 ± 1.2	74.8 ± 1.5
HGNN	87.8 ± 0.8	83.5 ± 1.1	78.2 ± 1.4
Dynamic-HGNN	89.2 ± 0.7	85.6 ± 1.0	81.4 ± 1.3
CHGNN	91.5 ± 0.6	88.3 ± 0.8	85.2 ± 1.2
ST-HGNN	92.7 ± 0.5	90.1 ± 0.7	87.6 ± 1.0
**H^2^GNN**	**95.2 ± 0.3**	**93.4 ± 0.5**	**91.5 ± 0.7**

**Table 5:** Noise robustness evaluation on CWRU dataset with varying SNRs. Values are mean accuracy (%).

Method	SNR = −4 dB	SNR = −2 dB	SNR = 0 dB	SNR = 2 dB	SNR = 4 dB
CNN	68.4	75.2	80.5	84.1	86.8
ST-HGNN	82.5	87.1	91.8	94.5	96.2
H^2^GNN	86.3	91.5	95.7	97.8	98.9

**Table 6:** Hyperparameter sensitivity on CWRU accuracy (%).

		Feature Dimension *d*			
		32	64	128	256
*K*	3	99.45	99.58	99.65	99.68
	5	99.52	99.71	99.87	99.82
	7	99.48	99.65	99.81	99.76
	10	99.31	99.52	99.70	99.64

**Table 7:** Efficiency comparison between H^2^GNN and the baseline ST-HGNN on the CWRU dataset. Throughput is measured as processed samples per second during inference on an NVIDIA RTX 3090 GPU.

Model	Parameters (M)	FLOPs (G)	Memory (MB)	Throughput (Samples/s)
ST-HGNN	1.25	0.42	845	526
H^2^GNN	1.38	0.58	1024	434

## Data Availability

The dataset analyzed in this study is publicly available. The Case Western Reserve University (CWRU) Bearing Dataset is available at https://engineering.case.edu/bearingdatacenter/download-data-file (last accessed on 1 May 2026). The Machinery Failure Prevention Technology (MFPT) Bearing Dataset is available at https://www.kaggle.com/datasets/emperorpein/mfpt-fault-datasets (last accessed on 1 May 2026). No new proprietary dataset was generated in this study. Additional implementation details needed to reproduce the reported protocol are provided in the Section 3.

## Abbreviations

The following abbreviations are used in this manuscript:

H^2^GNN	Hyperbolic Hypergraph Neural Network
HGNN	Hypergraph Neural Network
GCN	Graph Convolutional Network
CNN	Convolutional Neural Network
CWRU	Case Western Reserve University
MFPT	Machinery Failure Prevention Technology
IR	Inner Race
OR	Outer Race
BA	Ball Fault
t-SNE	t-Distributed Stochastic Neighbor Embedding
SVM	Support Vector Machine
ReLU	Rectified Linear Unit
ST-HGNN	Spatial-Temporal Hypergraph Neural Network
CHGNN	Contrastive Hypergraph Neural Network
